# Underexplored Ligand‐Binding Features of FabI From *Staphylococcus aureus* and *Escherichia coli*: A Comparative Pharmacophoric Modeling and Surface Mapping Approach

**DOI:** 10.1002/cmdc.70410

**Published:** 2026-07-31

**Authors:** Pedro Tenório T. F. Leite, Lucas H. S. Ocarino, Gabriel C. Veríssimo, Philipe O. Fernandes, Dara Fernanda Pereira, Fernanda Kelly M. e Oliveira, Alberto Marbán‐González, José L. Medina‐Franco, Mateus Sá M. Serafim, Thales Kronenberger, Vinícius G. Maltarollo

**Affiliations:** ^1^ Departamento de Produtos Farmacêuticos Faculdade de Farmácia Universidade Federal de Minas Gerais (UFMG) Belo Horizonte Minas Gerais Brazil; ^2^ Centro Universitário UNA Belo Horizonte Minas Gerais Brazil; ^3^ Departamento de Análises Clínicas e Toxicológicas Faculdade de Farmácia Universidade Federal de Minas Gerais (UFMG) Belo Horizonte Minas Gerais Brazil; ^4^ DIFACQUIM Research Group Department of Pharmacy School of Chemistry Universidad Nacional Autónoma de México Mexico City Mexico; ^5^ School of Pharmacy Faculty of Health Sciences University of Eastern Finland Kuopio Finland; ^6^ Interfaculty Institute of Microbiology and Infection Medicine (IMIT) University of Tübingen Tübingen Germany; ^7^ Partner‐Site Tübingen German Center for Infection Research (DZIF) Tübingen Germany

**Keywords:** antibacterials, FabI, molecular dynamics, molecular modeling, pharmacophore applications

## Abstract

The rapid spread of antimicrobial resistance, particularly among pathogens such as *Staphylococcus aureus* and *Escherichia coli*, highlights the urgent need for novel antibacterial agents with new mechanisms of action. The bacterial enoyl‐acyl carrier protein reductase (FabI), an essential enzyme in fatty acid biosynthesis, represents a promising target for narrow‐spectrum antimicrobials. This study aimed to define consensus pharmacophore models and interaction profiles for FabI through an integrated computational approach. ConPhar and FTMap analyses were applied to experimental structures and molecular dynamics (MD) simulations, while protein–ligand interactions from crystallographic complexes were evaluated using PLIP. Results revealed a conserved binding core involving residues Y156/Y157 and A95 in both species. We also assessed the reliability of residues located in flexible regions by comparing MD snapshots and experimental structures, as well as the contribution of inhibitor–cofactor interactions. Surface mapping identified Y146/Y147 as a key residue, consistent with its reported role in resistance mutations. Additionally, residues I200 (*E. coli*), V201 (*S. aureus*), and F203/F204 were identified as potential unexplored interaction sites. Finally, validated consensus pharmacophore models were proposed for future virtual screening and inhibitor design.

## Graphical abstract

Antimicrobial resistance highlights the urgent need for novel FabI inhibitors. Using an integrated computational approach combining ConPhar pharmacophore modeling, FTMap surface mapping, and PLIP interaction profiling across experimental structures and structures from MD simulations, we identified a conserved binding core (Y156/Y157, A95) and unexplored hotspots (I200, V201, F203/F204). Validated consensus pharmacophore models were proposed for future virtual screening efforts.

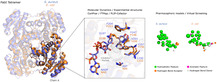

## 1 Introduction

The search for new drugs is a complex, onerous, and extensive process, and when it comes to antibacterials, urgency is the main factor that characterizes their development. The increase of antimicrobial‐resistant bacterial strains is a global health threat projected to cause 10 million deaths per year by 2050 [1]. According to the World Health Organization (WHO), antimicrobial resistance (AMR) was estimated to be directly responsible for 1.27 million deaths in 2019 and associated with a total of 4.95 million. In this scenario, *Escherichia coli* and *Staphylococcus aureus* are the two leading pathogens associated with mortality, with an increasing number of resistant strains, such as methicillin‐resistant *S. aureus* (MRSA), which caused more than 100,000 deaths in 2019 [2]. Thus, these species are frequent targets of drug discovery campaigns [3, 4].

In the search for novel bactericidal and bacteriostatic compounds, the bacterial enzyme NAD(P)H‐dependent enoyl‐ACP reductase (FabI) stands out as a promising target. FabI is a key component of the type II fatty acid biosynthesis (FAS‐II), which is essential in many microorganisms and fundamentally distinct from the polyprotein mammalian FAS‐I pathway [5]. This divergence makes the FAS‐II enzymes, especially FabI, attractive for selective antimicrobial development. FabI catalyzes the reduction of the enoyl‐ACP group to acyl‐ACP. Although FabV, FabK or FabL enzymes can also carry out this step in some microorganisms, FabI is solely responsible for the reaction in bacteria such as *S. aureus* and *E. coli* [6, 7]. Additionally, FabI inhibitors exhibit broad‐spectrum activity and can act against *S. aureus*, *S. epidermidis*, *Bacillus anthracis*, *B. cereus*, *E. coli*, *Pseudomonas aeruginosa*, *and Mycobacterium tuberculosis*, as well as *Plasmodium falciparum* [8]. Resistance against FabI inhibitors, however, is also a concern, and a deeper understanding of the inhibitor–protein interactions and structural adaptability is needed to aid the design of new compounds with reduced resistance potential [9]. For instance, Hafkin et al. [10] reported that the *S. aureus* FabI (herein termed *Sa*FabI) inhibitor AFN‐1252 (also called Debio‐1452 and here called AFN) has low propensity to select resistance. Conversely, Yao et al. [11] have previously pointed to an in vitro resistance by missense mutations associated with the selective pressure of AFN, such as M99T, which increased inhibition constant (*K*_*i*_) from 4 to 69 nM.

**FIGURE 1:**
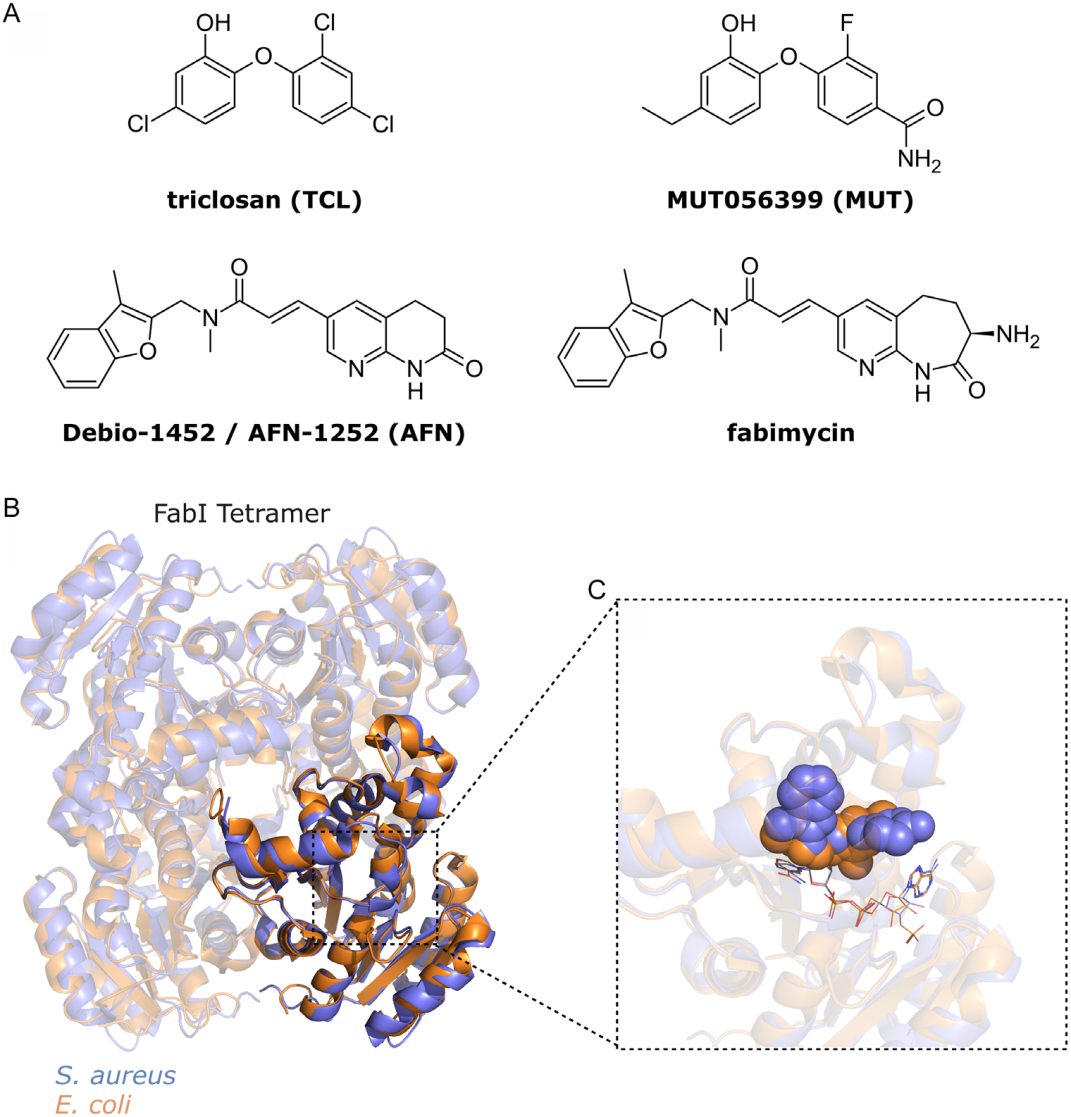
Chemical structures of relevant FabI inhibitors: triclosan (TCL), MUT056399 (MUT), AFN‐1251 (AFN) and fabimycin (A). Tetrameric structure of FabI from *S. aureus* (purple) and *E. coli* (orange) (B). Close view of ligand binding site (represented in spheres of AFN, purple, and TCL, orange) next to the cofactor site (represented as lines structures) (C).

The current clinical antibacterial drug candidates against FabI (AFN, triclosan [TCL], MUT056399 [MUT], and fabimycin, Figure 1A) are highly potent, usually occupying the catalytic site close to its cofactor (Figure 1B,C), but they are narrow‐spectrum, targeting primarily Gram‐positive bacteria (*S. aureus*). They can be further developed to spare the human gut/skin microbiome and commensal staphylococci, such as *S. lugdunensis* and *S. epidermidis* [12], but is not yet exploited to target Gram‐negative WHO bacterial priority pathogens such as *E. coli*, *Shigella* spp., *Salmonella* spp. or *Klebsiella pneumoniae*. Gram‐negative promiscuous efflux pumps easily export certain FabI inhibitor scaffolds (e.g., classic diphenyl ethers). Hence, we need novel chemotypes engineered specifically to overcome these limitations. A recent example is fabimycin, a novel scaffold structurally tailored to overcome Gram‐negative membrane permeation issues, which showed efficacy in vivo against drug‐resistant Gram‐negative bacteria infections in mouse models [12]. However, due to its prominent zwitterion character, the diphenyl ether scaffold is extremely prone to serum protein binding (i.e., significantly lowering the free drug concentration) and has rapid metabolic inactivation via liver glucuronidation/sulfation.

Considering the current scarcity of drug candidates, the identification of new bioactive compounds is crucial and can benefit from extensive molecular databases in virtual screening (VS) campaigns. Pharmacophore‐based VS is particularly valuable in drug discovery, based on defining a three‐dimensional model of essential molecular characteristics that describe target–ligand interactions and elicit a desired biological response [13]. Our group has extensively employed different computational approaches to discover new FabI inhibitors. Initially, hologram quantitative structure–activity relationship (HQSAR) models were employed for the VS of active compounds against *Sa*FabI [14]. These models were then adapted for *E. coli* FabI (herein termed *Ec*FabI) via a systematic comparison integrating 2D chemical similarity, molecular docking, and molecular dynamics (MD) simulations [15]. This workflow facilitated the discovery of three novel compounds active against multiple susceptible and resistant *S. aureus* strains, including clinical isolates [16]. Furthermore, these computational approaches enabled the elucidation of the potential mechanism of action for a series of 1,3‐bis(aryloxy)propan‐2‐amines exhibiting antibacterial activity [17].

To further predict *Sa*FabI inhibitory activity, machine learning (ML) models were developed using diverse algorithms, including decision trees, random forests, multilayer perceptrons, k‐nearest neighbors, naïve Bayes (NB), and support vector machines [18]. These predictive models were subsequently expanded using graph convolutional networks [19]. Both studies incorporated structure–activity relationship (SAR) analyses to identify key structural features of the ligands driving biological activity, providing a framework for the rational design and optimization of inhibitors. More recently, advanced ML models were proposed for *S. aureus* and MRSA, aiming to identify new antibacterial compounds via VS and experimental validation. This combined approach yielded six active compounds with minimum inhibitory concentrations (MIC) as low as 12.5 μM [20].

Furthermore, MD simulations have proven critical in detailing the effects of various inhibitor classes on the tetramerization of *E. coli* and *S. aureus* FabI, successfully identifying key regions for catalytic site inhibition and indicating computational filters for drug design [15, 21]. To overcome the high computational costs of applying MD to hundreds of candidates, integrating pharmacophore or ML‐based VS with subsequent MD simulations can effectively optimize resource allocation while preserving in silico predictive accuracy, thereby reducing the number of compounds requiring synthesis and in vitro validation. While FabI is under extensive study, a comprehensive consensus understanding its essential pharmacophoric features across diverse inhibitors and dynamic conformational states remains elusive. To fill this gap, we herein propose a consensus pharmacophore for *Sa* and *Ec*FabI enzymes, mapping the protein's interaction surface and identifying residues consistently involved in ligand binding, as well as those with potential for interactions not yet described.

## 2 Computational Methods

### 2.1 Dataset Retrieval

The dynamic nature of FabI is particularly relevant due to its multiple oligomeric states, where subunit interactions can influence both inhibitor binding and enzymatic function [21]. Therefore, we analyzed data from two distinct sources. The first was *S. aureu*s and *E. coli* experimental structures from the Protein Data Bank (PDB) [22], which are summarized in Table 1. Relevant ligand structures from those PDB structures are illustrated in Figure S1. The second was previously published MD simulations, comprising the FabI tetramer in complex with the inhibitors TCL, AFN, and MUT (five replicas of 3 μs each with a total of 5 μs for each system) [21]. The simulations were performed using Desmond with the OPLS4 force field, explicit TIP3P water solvation, and Na^+^/Cl^−^ counterions to neutralize the system in a cubic box with a buffer distance of 13 Å from any protein atom. Each system was clustered based on the root mean square deviation of ligand site residues to obtain conformational diversity of the binding pocket, using hierarchical clustering analysis with default parameters implemented on Maestro (v2024v3 ‐ using the script trj_cluster.py with a cut‐off of 1 Å for the protein's backbone). One representative structure from each of the top three most populated clusters was selected.

**TABLE 1:** Analyzed experimental structures of FabI in complexes with different inhibitors.

PDB	Resolution, Å	Model assembly	Co‐factor	Ligand	Species
3GR6 [23]	2.28	Tetramer	NADP	Triclosan	*S. aureus*
4ALL [24]	2.80	Tetramer	NADP	Triclosan	
4FS3 [9]	1.80	Tetramer	NADPH	AFN‐1252	
4NZ9 [25]	2.30	Dimmer	NADP	Benzimidazole derivative	
6TBB [26]	2.45	Tetramer	NADPH	Kalimantacin A	
6TBC [26]	2.55	Tetramer	NADPH	Kalimantacin B	
1C14 [27]	2.00	Dimer	NAD	Triclosan	*E. coli*
1D8A [28]	2.20	Dimer	NAD	Triclosan	
1DFH [29]	2.20	Dimer	NAD	Thieno‐diazaborine derivative	
1I2Z [30]	2.80	Dimer	NAD	BRL‐12 654	
1I30 [30]	2.40	Dimer	NAD	SB385826	
1LX6 [31]	2.40	Dimer	NAD	Benzamide derivative	
1LXC [31]	2.40	Dimer	NAD	Acrylamide derivative	
1MFP [32]	2.33	Dimer	NAD	SB611113	
1QG6 [33]	1.90	Tetramer	NAD	Triclosan	
1QSG [34]	1.90	Tetramer	NAD	Triclosan	
1DFG [29]	2.50	Dimer	NAD	Benzodiazaborine	
4CV2 [35]	1.80	Dimer	1,4‐Dihydronicotinamide adenine dinucleotide	CG400549	
4CV3 [35]	1.95	Dimer	1,4‐Dihydronicotinamide adenine dinucleotide	PT166	
4D46 [36]	2.00	Dimer	NAD	Benzonitrile derivative	
4JQC [37]	2.80	Dimer	NAD	AFN‐1252	
4JX8 [38]	3.20	Dimer	NAD	AEA16	
5CG1 [39]	2.07	Dimer	NAD	Carbamoylated benzodiazaborine inhibitor 14b	
5CG2 [39]	2.11	Dimer	NAD	Thiocarbamoylated benzodiazaborine inhibitor 35b	
7UM8 [12]	1.70	Dimer	NAD	(R, E)‐3‐(7‐amino‐8‐oxo‐6,7,8,9‐tetrahydro‐5H‐pyrido[2,3‐b]azepin‐3‐yl)‐N‐methyl‐N‐((3‐methylbenzofuran‐2‐yl)methyl)acrylamide	
7UMW [12]	1.54	Dimer	NAD	Fabimycin	

All FabI structures were retrieved from the PDB until April, 2026 (provided as an extended dataset in the Zenodo 10.5281/zenodo.19693991 together with filtered metadata). Structures were treated according to Maltarollo et al. [21] and multimers were split into monomers and aligned by their backbone using Maestro (2024v3). To evaluate structural similarities, we performed an all‐against‐all pairwise structure comparison of FabI PDB structures. Analyses were carried out considering the Template Modeling (TM)‐score computed by the TM‐align algorithm implemented in the US‐align standalone, using the monomeric structure alignment option [40]. TM‐align calculates two TM‐scores normalized by the length of each pair of structures. Thus, the mean of the two TM‐scores was calculated to represent each protein pair, and the resulting matrix of pairwise distance values was used for a multidimension scaling (MDS) analysis.

### 2.2 Pharmacophoric Modeling

Pharmacophoric modeling was performed using the ConPhar algorithm [41] with default parameters, which calculates potential ligand–receptor interactions and outputs details about their type and frequency, including heatmaps for each interaction type and a final consensus pharmacophore model. The results were visualized using PyMOL [42]. The Python libraries “pandas,” “plotly,” “seaborn,” and “scikit‐learn” were considered for data processing and visualization [43].

The input protein structures were first categorized by species into two groups: *S. aureus* (Group 1) and *E. coli* (Group 2). Within each group, structures were further classified into subgroups based on their origin. The PDB subgroup consisted of experimentally determined structures without any mutations. The MD subgroup comprised representative conformations derived from MD simulations, retrieved from Maltarollo et al. [21]. For this subgroup, chains corresponding to each of the three proteins were structurally aligned and subsequently isolated for ConPhar analysis. The combined subgroup included structures from both PDB and MD and was used to evaluate the consistency of the results across all structural sources. Detailed information on the structures included in each subgroup and the corresponding number of structures is summarized in Table 2.

**TABLE 2:** Pharmacophoric groups and tests details.

Group 1 (*S. aureus*)	Number of structures	Description
PDB	6	*S. aureus* structures available in the PDB with cocrystalized ligands
MD	12	MD frames with ligands: TCL, AFN, and MUT
Combined	18	Combination of PDB and MD inputs
**Group 2 (*E. coli*)**	**Number of structures**	**Description**
PDB	20	*E. coli* structures available in the PDB with cocrystalized ligands
MD	12	MD frames with ligands: TCL, AFN, and MUT
Combined	32	Combination of PDB and MD inputs

Finally, we built pharmacophore models based on each of the combined groups of *S. aureus* and *E. coli*, considering a combination of one or more of the four interaction types represented by spheres: aromatic, hydrophobic, hydrogen acceptor, and hydrogen donor. Each model was selected based on the overall prevalence of specific interaction types and the spatial clustering of interaction points. In other words, we considered positions identifying central spheres within dense clusters of the same type of interaction or by prioritizing larger spheres.

### 2.3 Consensus Pharmacophore Model Virtual Screening Validation

The consensus pharmacophore models built were evaluated in virtual screening (VS) performance with the receiver operating characteristic area under the curve (ROC AUC), and the Boltzmann‐enhanced discrimination of receiver operating characteristic (BEDROC), with α values adjusted for enrichment factors (EF) of 1%, 3%, 5%, and 10%, according to Truchon and Bayly [44]. This assessment was performed with the dataset of inhibitors and decoys from Veríssimo 2023 [45], comprising 177 actives and 11,467 decoys for *S. aureus* and 73 actives and 3,927 decoys for *E. coli*. Using OMEGA 5.1.0 [46], we created a set of 30 lowest energy conformers per compound, thus increasing the number of possible conformations to be filtered in the VS validation. The screening was performed using vROCS 3.9.0 [47] considering default parameters. For each model and each vROCS output metric, all ROC AUC and BEDROC values were calculated to indicate the overall performance in true active inhibitor versus decoy discrimination.

### 2.4 Hotspot Mapping

Potential binding hotspot residues on the FabI surface were predicted and characterized using the FTMap server [48]. The representative structures from the MD simulations were analyzed to identify potential binding pockets correlated with the spatial clustering of the ligands. The initial output contained numerous probe clusters in the high‐affinity cofactor binding site (Figure 1). Nonetheless, to focus our analysis on the inhibitor binding site, these cofactor site clusters were manually curated and removed from subsequent analysis. The remaining clusters were analyzed with the protein–ligand interaction profiler (PLIP) to extract the specific residues and types of interactions [49]. This data was compiled with Python programming language to gather the results across all the selected positions, organizing the data by species.

### 2.5 Protein–Ligand Interaction Profiles of Experimental and MD Structures

The protein–ligand interactions of experimental and MD structures were analyzed with PLIP, contextualizing the FTMap and ConPhar results. Each protein structure was treated to remove its water molecules, keeping only chain A and the inhibitors. For the experimental structures, a modified version of PLIP (available on GitHub) [50, 51] was employed to capture interactions between the inhibitors and the cocrystallized cofactor, which might be crucial for binding affinity and inhibitory activity. The interaction profiles from these analyses were compiled similarly to the FTMap results by showing interaction count for each residue and interaction type.

## 3 Results

### 3.1 FabI has Low Diversity on Experimentally Derived Structures

**FIGURE 2:**
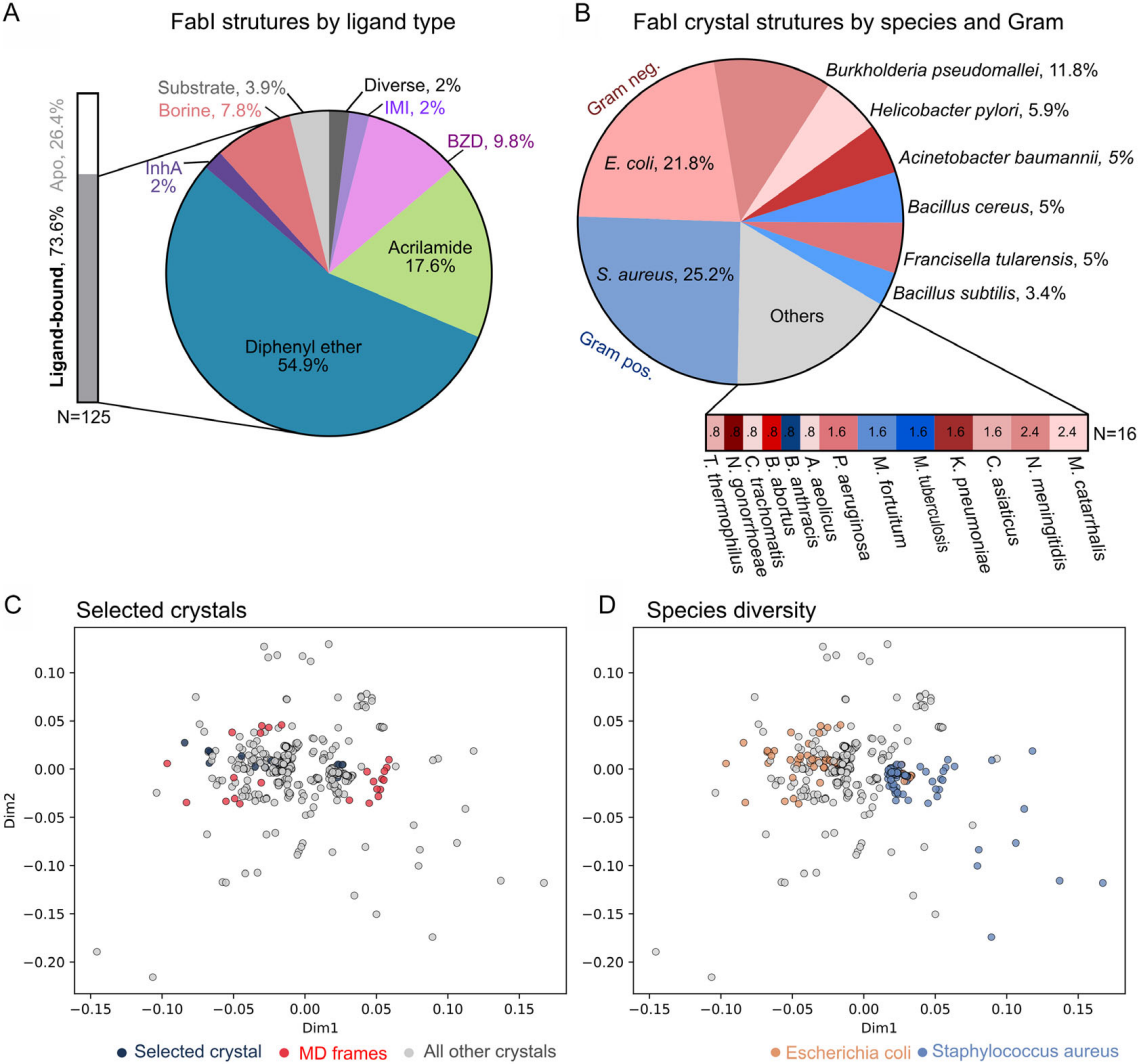
FabI structural diversity. Classification of FabI crystal structures according to their bound ligands (A) or species (B). Multidimensional analyses based on structural alignment of all available crystals from FabI colored by the selected crystals for pharmacophoric models versus MD selected frames (C) or by species (D). Other relevant scatter plots are available as Figure S2.

We started by systematically analyzing all available FabI experimental structures available until April, 2026 (see extended dataset on 10.5281/zenodo.19693991). Those structures were retrieved from the PDB database using FabI as keyword and filtering by EC keywords. Among the retrieved 125 unique models, 26.4% were apostructures, which we defined as only having cofactors as ligands, and the other 73.6% represented inhibitor‐bound experimental structures (Figure 2A). Among the inhibitor‐bound ones, more than half are bound to diphenyl ether compounds, that is, TCL and TCL analogs, distantly followed by molecules with an acrylamide core (see Figure S2 for structural differences induced by ligands). Those structures belong to a range of relevant Gram‐positive and negative bacteria (Figure 2B) with *S. aureus* and *E. coli* structures clearly separating into two clusters on MDS analyses, which suggests they are structurally distinct (Figure 2D).

Most selected crystals (blue dots, Figure 2C) fall into a single relevant cluster in the center of the MDS plot with other structures that do not contain inhibitors (Apostructures) and are, therefore, not relevant for pharmacophores relevant structures (Figure S2). Selected MD simulation frames (red dots) occupy an overlapping structural space as the selected crystals—which suggests that they might have little effect on the pharmacophore modeling.

### 3.2 Pharmacophoric Modeling and Virtual Screening Performance

**FIGURE 3:**
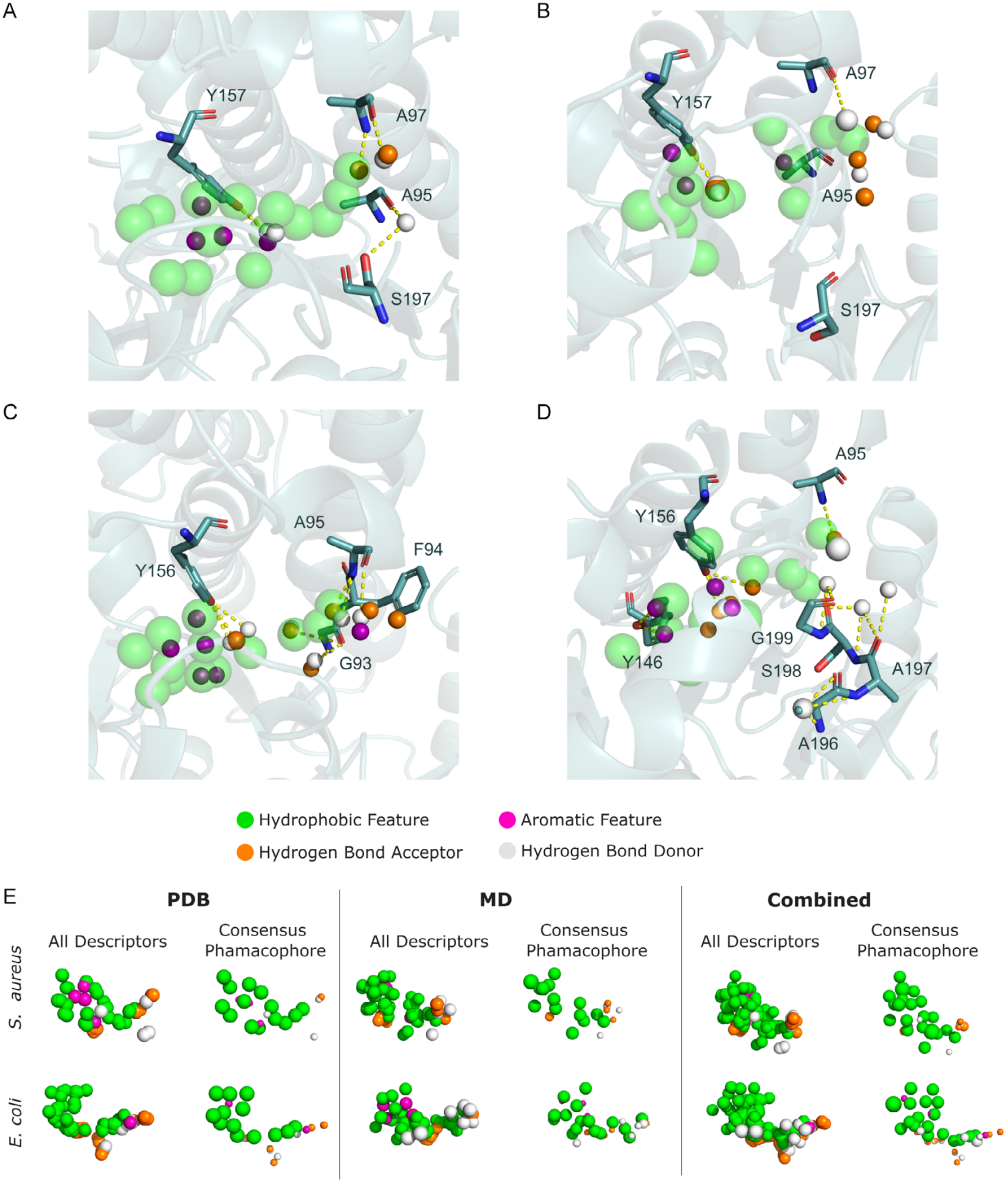
Model from MD simulation data; model combining PDB structures and MD simulation data. (A) Consensus pharmacophore generated from the PDB *S. aureus* group (PDB 6TBB). (B) Consensus pharmacophore generated from the MD *S. aureus* group. (C) Consensus pharmacophore generated from the PDB *E. coli* group (PDB 4JQC). (D) Consensus pharmacophore generated from the MD *E. coli* group; (E) pharmacophoric models of FabI inhibitors *S. aureus* (group 1) and *E. coli* (group 2): model from PDB structures.

We analyzed the pharmacophoric models from the PDB structures, which highlighted a high density of hydrophobic features for both species covering most of the binding pocket, close to aromatic interactions. This is consistent with the fatty acid nature of FabI's natural substrates. Although hydrophobic features exhibited a greater deviation, the polar interactions were more consistently positioned near residues A97/A95 and Y157/Y156 (Figure 3A,C).

Next, incorporating the MD's conformational variability yielded models with some differences from the PDB data. While the core pattern of a hydrophobic region flanked by polar features was conserved, these models displayed higher positional deviation of polar descriptors, as observed by their larger spheres (Figure 3B,D). Furthermore, the *E. coli* model revealed additional aromatic interaction sites. These discrepancies highlight the impact of protein flexibility and are likely attributable to the difference in chemical diversity when compared to the set analyzed from the PDB structures assessed for the pharmacophoric modeling. Then, when combining both models for each species, the integration of both sets of structures provided a more comprehensive view (Figure 3E). As expected, the increased structural diversity resulted in models with a higher number of features and greater positioning deviation. Notably, the *E. coli* model also showed a region of aromatic interactions that was only present in the PDB pharmacophoric model, arguably due to its ligand diversity.

Mapping the polar features of consensus pharmacophore models onto the protein structure revealed key details in the binding site residues. *S. aureus* PDB pharmacophoric model (Figure 3A) highlighted residues A95 and S197 interacting via hydrogen bonds with the backbone's carbonyl group and the side chain's hydroxyl group, respectively. In addition, the A97 backbone shows both hydrogen bond interactions, while the Y157 phenol hydroxyl group can act as a hydrogen bond acceptor (HBA) and donor. Similarly, the model obtained from MD data (Figure 3B) had the polar features of A97 and Y157 consistent with the PDB's pharmacophoric model. Conversely, A95 shifted, and the S197 side‐chain was notably facing away from the pharmacophoric model. S197 is part of a flexible loop, and its movement in the MD simulations underscores the limitation of relying solely on rigid experimental structures. This comparison showcases the fundamental importance of Y157 and A97 for inhibition across dynamic conformational states, making them prime targets for robust inhibitor design. Additionally, the *E. coli* PDB pharmacophoric model highlighted A95 and Y156 (Figure 3C) positioned similarly to their *S. aureus* counterparts, whereas the newly mapped G93 could exhibit hydrogen bond interactions. Similarly, both *E. coli* models showed Y157 and A97 interactions, whereas the flexible loop containing A196, A197, S198, and G199 was seen only in the MD‐derived model (Figure 3D). Last, the consensus pharmacophore extracted the most likely interactions from a broader set of possible descriptors, and the sphere sizes indicate a deviation of its position in the three‐dimensional space (Figure 3E).

**FIGURE 4:**
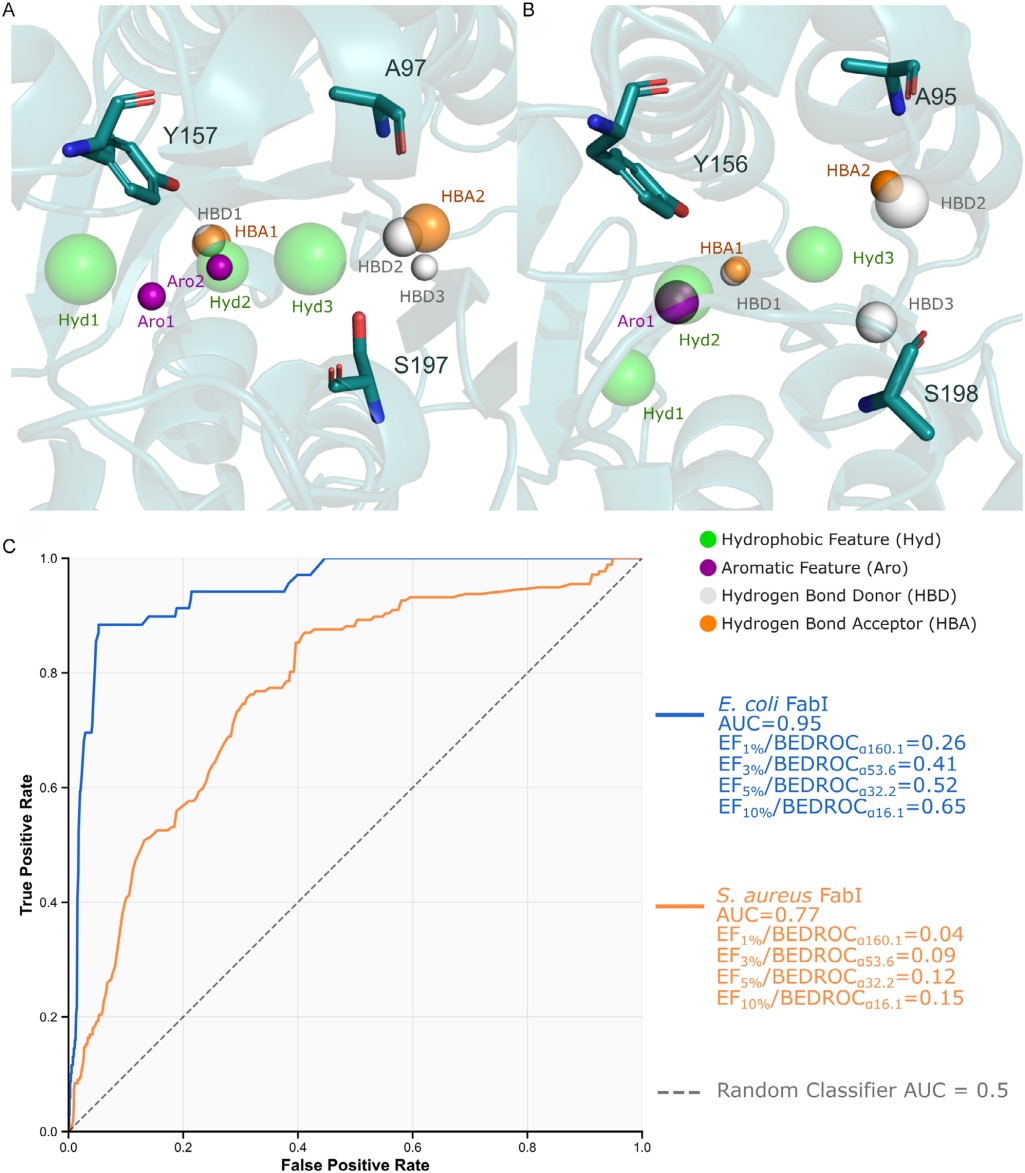
Consensus pharmacophoric models of FabI inhibitors. (A) *S. aureus* and (B) *E. coli* models (deepteal) are represented based on PDBs 6TBB and 4JQC, respectively. (C) Best performing ROC curves ranked by TverskyColor, with their respective ROC AUC values, and enrichment factors at 1%, 3%, 5% and 10%.

Subsequently, to perform the VS validation, we selected a set of consensus interaction points, represented by spheres, to build a consensus pharmacophore model for both the *S. aureus* and *E. coli* FabI enzymes (Figure 4). At this step, we removed redundant features (i.e., two or more overlapping spheres or too‐close points and kept just one feature at centroid) aiming for a simpler pharmacophoric model. For *S. aureus*, there were two aromatic, three hydrophobic, two HBA and three hydrogen bond donor (HBD) spheres (Figure 4A), while for *E. coli* there was one aromatic, three hydrophobic, two HBA and three HBD spheres (Figure 4B). For both models, the best‐performing metric was Tversky Color (Figures 4C and S3), which showed high discrimination for *E. coli* (AUC 0.947) and moderate discrimination for *S. aureus* (AUC 0.77). A possibility for this discrepancy is that the larger sampled structural data for the *E. coli* PDB group (Table 2) resulted in a more accurate model. Additionally, *E. coli* ROC curves had considerably higher values for early recognition, as shown by EF values at 1%, 3%, 5%, and 10%; that is, the model had a higher tendency to rank actives (true inhibitors) earlier in the screening set. Of note, the latter's dataset of inhibitors and decoys is ∼65% smaller (*n* = 4000 compounds) compared to the *S. aureus* set (*n* = 11,544 compounds), which could have a less precise estimate of the AUC. The confidence intervals on a smaller set could arguably be wider, that is, the score would be more likely to reflect a real performance of the models when assessing a broader chemical space. For instance, having less diverse scaffolds might make a model appear to have superior performance because it is overfitting to the limited diversity of that set [52].

### 3.3 Surface Mapping

**FIGURE 5:**
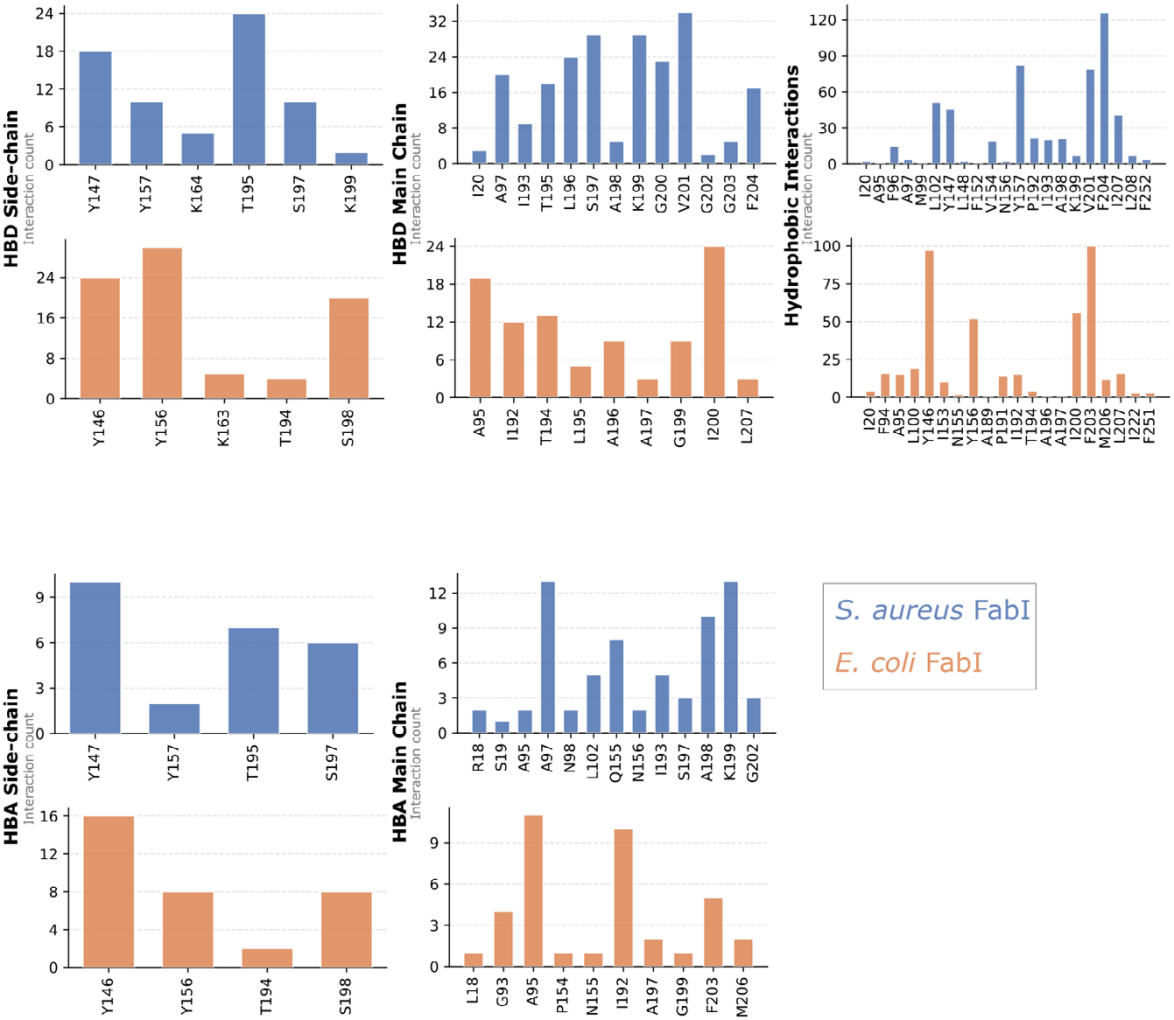
Residue interaction counts for each type of interaction found for FTMap probes analyzed by PLIP for *S. aureus* (blue) and *E. coli* (orange). Hydrogen bond donor and acceptor interactions count are divided by side‐ and main chains (backbone), alongside hydrophobic interactions count.

Our FTMap predictions identified clusters at the cofactor‐binding site (Figure S4), which were excluded from subsequent analyses as described in the Methods section. A summary of the interactions observed in the inhibitor‐binding analysis is presented in Figure 5. For side‐chain HBD interactions, the prominent mapped residues in *S. aureus* were Y195, Y147, Y157, and S198, while Y147, Y157, and S197 in *E. coli*. Consistent with the structural similarity between FabI from the two species, the interaction patterns were conserved. For example, *S. aureus* Y195, the most frequently mapped residue for side‐chain HBA interactions, corresponded to the mapped *E. coli* residue Y194. Overall, the same set of residues was mapped for side‐chain HBD interactions in both species, with the exception of K199, which was identified only in *S. aureus*. On average, backbone HBD interactions were more frequent in *S. aureus* than in *E. coli*, with V201 and I200 being the most frequently mapped residues.

Regarding side‐chain HBA interactions, the mapped residues in *S. aureus* were Y147, Y195, and S197, while Y146, Y156, and S198 in *E. coli*. The same pattern observed for side‐chain HBD interactions was also found for side‐chain HBA, with corresponding residues mapped in both proteins. As for the backbone HBA interactions, A97 and K199 were mapped in *S. aureus*, whereas A95 and I192 were identified in *E. coli*. Finally, hydrophobic interactions were mostly involved with F204, Y157, and V201 in *S. aureus*, and F203 and Y146 in *E. coli*.

### 3.4 Protein–Ligand Interaction Profiles of Experimental and MD Structures

**FIGURE 6:**
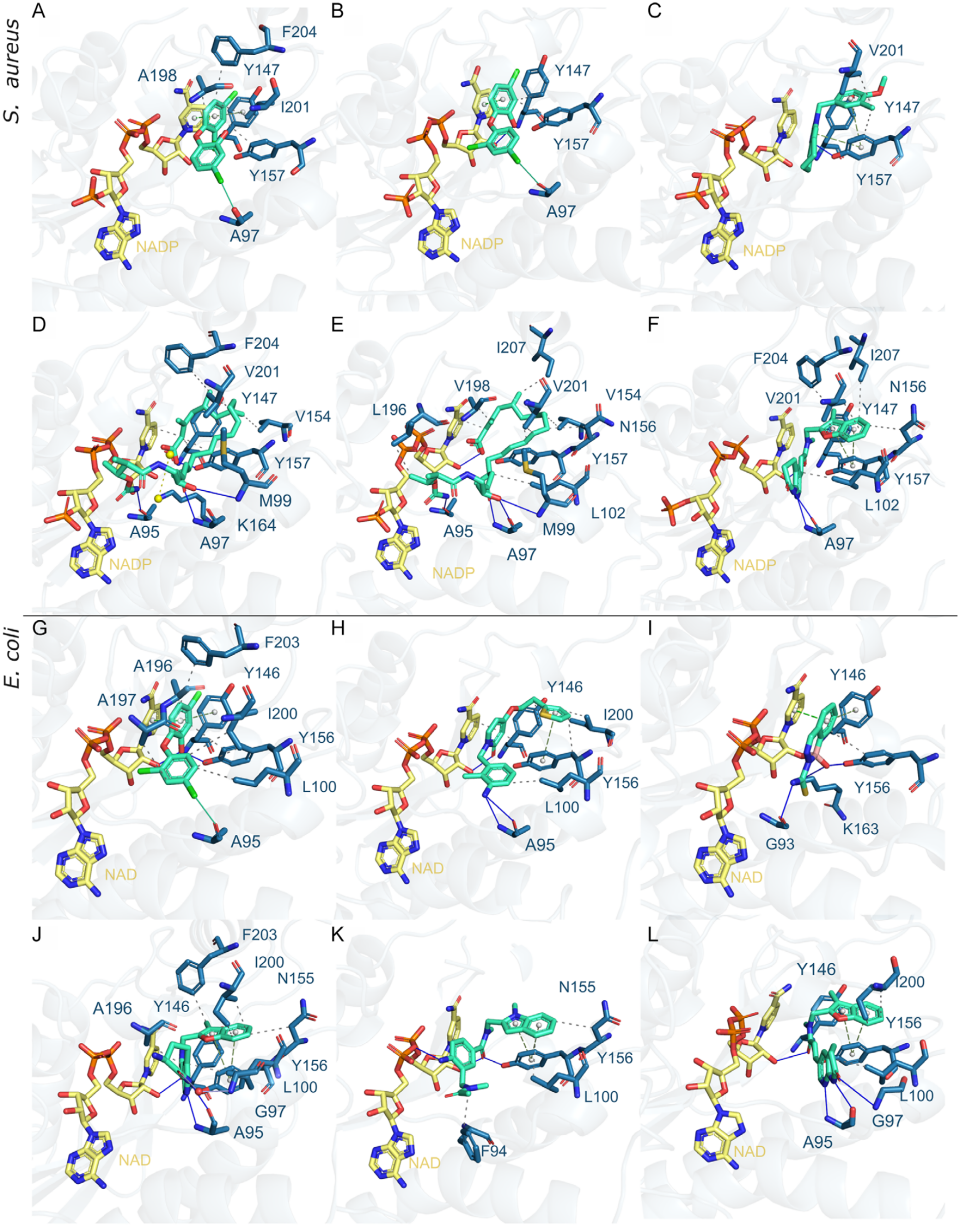
Protein–ligand interactions extracted with PLIP‐cofactor for the crystallized structures of *S. aureus* (A–F) and *E. coli* (G–L) FabI bound to inhibitors. (A) and (B), triclosan, PDBs 3GR6 and 4ALL, (C) benzoimidazole derivative, PDB 4NZ9, (D) kalimantacin A, PDB 6TBB, (E) kalimantacin B, PDB 6TBC, (F) AFN‐1252, PDB 4FS3. (G) triclosan, PDB 1D8A, (H) CG400549, PDB 4CV2, (I) thiocarbamoylated benzodiazaborine inhibitor 35b, PDB 5CG2, (J) Fabimicyn, PDB 7UMW, (K) benzamide derivative, PDB 1LX6, (L) AFN‐1252, PDB 4JQC. Carbon chains of amino acid residues are shown as dark blue sticks, while carbon chains of ligands are shown as green‐cyan sticks, and cofactors are depicted in light yellow. Hydrogen bonds are displayed as full blue lines, hydrophobic bonds as dashed gray lines, π‐stacking interactions as light green dashed lines, salt bridges as yellow dashed lines, and halogen bonds as full green lines.

The analyses performed with experimental structures and MD trajectories using PLIP served as the validation for the computational predictions. For *S. aureus*, residues A95, A97, Y147, and Y157 are predicted once again with high interaction counts, in agreement with both the FTMap and ConPhar results (Figures 6 and S5–S8). While minor variations in interactions were observed, arguably caused by distinct ligand poses such as TCL, which was validated in different orientations in PDBs 3GR6 and 4ALL (evidenced from the electron density maps, Figure S9), the central set of residues was consistently engaged in all structures. Notably, every inhibitor forms multiple interactions with the cofactor in *S. aureus* structures, with the exception of the benzamidazole derivative (Figure 6C). The main cofactor–inhibitor interactions found are the π‐stacking interactions for TCL (Figure 6A,B) and hydrophobic interactions involving the same aromatic ring (Figure 6D,E), as well as hydrogen bonds with the ribose hydroxyl and phosphate groups (Figure 6E,F). Interestingly, TCL forms a halogen bond with A95/A97 (Figure 6A,B,G), while most other inhibitors form a hydrogen bond (Figure 6D–F,H,J, L). The carboxylate groups of Kalimantacin A and Kalimantacin B are shown to interact with either K164 via a salt bridge (Figure 6D) or the cofactor's ribose hydroxyl (Figure 6E).

A parallel analysis of *E. coli* structures revealed a similar profile, with a highly conserved core involving residues A95, L100, Y146, Y156, and I200 (Figures 6G–I and S5–S8), which is consistent with our pharmacophoric models. Minor differences in interaction patterns between similar inhibitors, such as a water bridge formed between Fabimicyn and G97, which becomes a direct hydrogen bond in AFN‐1252's experimental structure, can be attributed to factors like solvation and subtle side‐chain rearrangements. These observations highlight the value of analyzing a broad ensemble of structures to distinguish robust and conserved interactions from more variable, less conserved ones. As for the cofactor, the same major interactions found for *S. aureus* were predicted in *E. coli*, even including the notable covalent bond with the inhibitor in PDB 5CG2 (Figure 6I).

**FIGURE 7:**
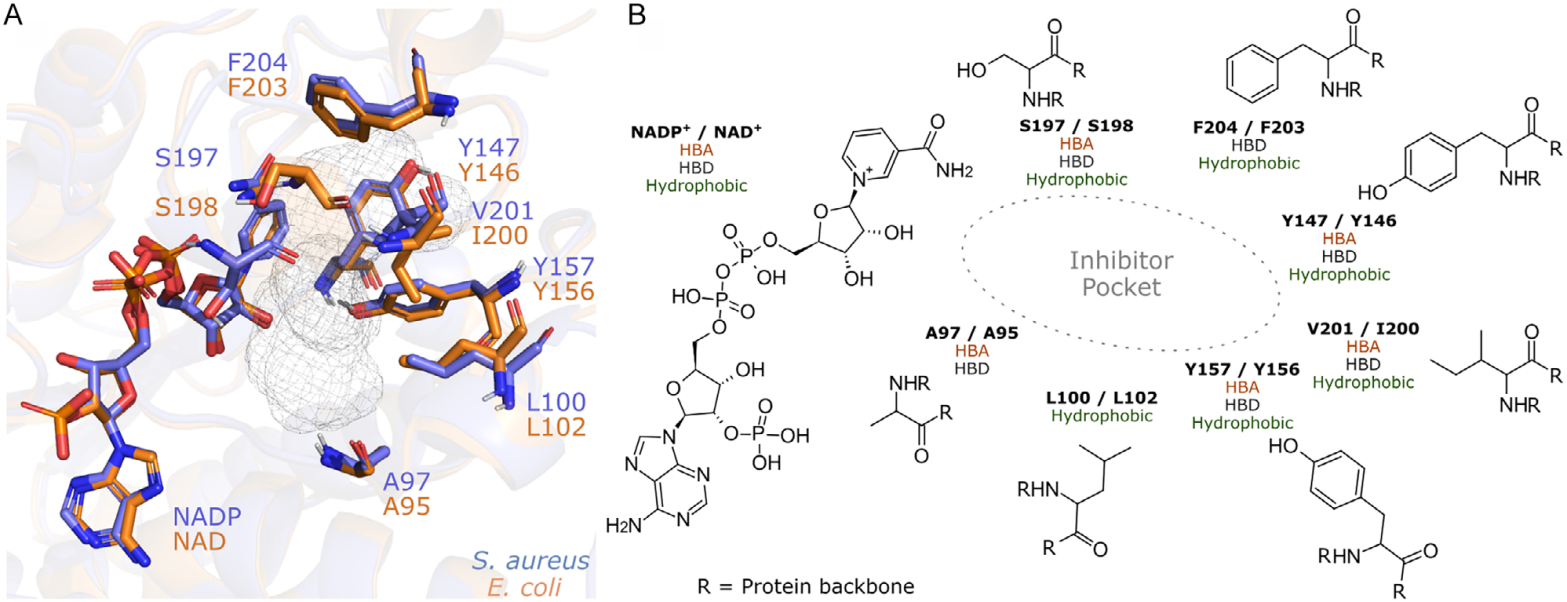
Proposed interacting residues to be explored based on pharmacophoric modeling and hotspot mapping for FabI inhibition. (A) Three‐dimensional representation, (B) two‐dimensional representation.

Finally, these findings reveal additional underexplored binding hotspots through the surface mapping and crystal analyses. Namely, the cofactor, L100/L102, I200/V201, F203/F204, and the conformationally flexible loop residues S197/S198 (Figure 7).

## 4 Discussion

The conserved interaction network identified by our analyses is supported by previously reported computational studies of FabI inhibition. For instance, A95/A97, Y146/Y147, and Y156/Y157 are key residues in the FabI active site essential for both catalysis and inhibition [14, 18, 53]. A95/A97 help recognize the natural enoyl‐ACP substrate and act as anchoring points for inhibitors such as AFN‐1252 and fabimycin via hydrogen bonds and hydrophobic interactions with the backbone [21, 36]. Because these inhibitors often target main chain atoms rather than side‐chain ones, targeting this region can be considered a useful strategy for minimizing mutations that confer resistance, although substitutions such as A95V can still confer resistance to compounds like triclosan [54]. Moreover, Y146/Y147 contributes to inhibitor binding through π‐stacking and hydrophobic interactions. The single point substitution Y147C is known to decrease kalimantacin activity against *S. aureus* [26]. In this study, either Y147C or M99T was responsible for increasing the compound MIC from 0.125 to 0.5 µg/mL against *S. aureus* RN4220 strain and up to 16 µg/mL when both substitutions are present in the mutated enzyme. In support of this notion, a 16‐fold increase in MIC values of triclosan from 0.25 to 4 µg/mL against the Y147C mutated *S. aureus* strain FM026 [55]. Additionally, Y147H can also promote reduced sensitivity of different *S. aureus* strains to triclosan, AFN‐1252, and CG400462 [9, 56]. Tyr156/Tyr157, on the contrary, is part of the conserved catalytic triad and is crucial for catalysis and stabilization of the enzyme–inhibitor complex through hydrogen bonding and π‐stacking interactions [57, 58]. Altogether, these residues define the structural and catalytic framework of FabI and are major determinants of inhibitor potency and potential resistance.

Different computational studies have also investigated the structural requirements for *Sa*FabI inhibition. For instance, HQSAR combined with molecular interaction field analyses, comparing FTMap fragment mapping, was able to indicate A95/A97, Y146/Y147, and Y156/Y157 as key inhibitor‐interacting residues from a single PDB structure [14], which is consistent with our current findings. These features are important for the identification of new inhibitors as potential antibacterial drug candidates. For instance, Henver et al. [59] identified a novel and potent benzimidazole‐derived class of *Francisella tularensis* FabI inhibitors combining 3D and electrostatic similarity analyses with known active compounds. The authors prioritized conserved recognition features shared across known inhibitor classes, notably an aromatic moiety that could form π‐stacking interactions with NAD+ nicotinamide ring and a HBA oriented toward Y156. Based on these observations, a VS was performed, which resulted in three hit compounds, and ultimately optimized analogs reaching submicromolar inhibitory activity. Similarly, a VS reported by Asse Junior et al. [16] employed 3D shape and electrostatic similarity models based on known FabI inhibitors to identify new antibacterial compounds against *S. aureus*, MRSA, and *E. coli*. Their evaluation of chemical feature contributions revealed that the presence of HBAs, corresponding to interactions with Y157 and A97, was important for improving their classification model's performance, which supports our pharmacophoric models, where various hydrogen bond‐forming residues were identified.

Recently, the structure‐guided optimization of AFN‐1252 against *Neisseria gonorrhoeae* was reported [60], achieving Debio 1453, a sub‐nanomolar inhibitor with potent activity against multidrug‐resistant clinical isolates. The authors optimized van der Waals interactions within the active site and promoted interactions with NADH through water‐mediated interactions, improving potency and limiting the potential for resistance to occur. Debio 1453 showed activity in vivo, evaluated in vaginal gonorrhoea murine models, while also being active against *S. aureus* *i*n vitro and in vivo. Combining molecular features from distinct FabI regions is also a valuable strategy to increase potency and selectivity. In this sense, it is also important to consider residues in their water molecule occupancies from different structures, especially the core‐targeted residues, such as A95/A97, Y146/Y147, and Y156/Y157 [21].

Based on these observations and our analyses, using consensus information to build pharmacophore models that summarize important structural features can arguably favor the identification of new FabI inhibitors from different chemical libraries. For instance there are recently designed chemical libraries (a total of 172,026 compounds) focused on FabI of *S. aureus*, which were built using transformation rules and ML models [61], as well as the potential FabI inhibition from natural products libraries [62]. Thus, we proposed a consensus pharmacophoric model and performed a VS validation, achieving moderate to high discrimination results (0.77 and 0.947). Considering the potential applications of these two models, we can compare their performance with similar VS reported in the literature. For example, Kronenberger et al. [15] reported a 3D similarity model only for *Ec*FabI inhibitors with an AUC‐ROC of 0.996. However, this study focused solely on TCL derivatives with a smaller dataset of 48 active compounds and 1728 decoys. Assé Jr. et al. [16] also reported 3D similarity models for *Sa*FabI inhibitors with AUC ranging from 0.945 to 0.951. In this second case, despite the limited dataset in comparison with the present data, the authors performed a VS and tested hit compounds against *S. aureus*, MRSA, and *E. coli*, finding true active compounds with MIC values as low as 15.6 μM. Interestingly, Liu and collaborators [63] reported ML models for *Sa*FabI inhibitors classification with external validations comparable to our pharmacophoric model (AUC ranging from 0.759 to 0.788), which resulted in a hit identified by VS, compound Y4, which inhibited FabI in enzymatic assays (IC_50_ of 9.83 μM).

Hevener et al. [59] also used a combination of 3D and electrostatic similarity models that achieved AUC ranging from 0.75 to 1.00, but using limited datasets having 14–33 compounds split by chemical classes. Still, the VS campaign identified a hit that showed enzymatic inhibitory activities, having an IC_50_ value of 27 μM against *Francisella tularensis* FabI. This hit was ultimately optimized with the synthesis of two new analog compounds, having IC_50_ values around 0.3 μM against the same enzyme. Taken together, our findings support a reasonable consensus pharmacophoric model for VS, which had comparable AUC values to other models and methods reported in the literature.

## 5 Conclusions

This study integrated ligand‐based and structure‐based computational approaches to define the essential molecular interactions of known inhibitors of the FabI enzyme in *S. aureus* and *E. coli*, as well as to suggest new possible interactions that can be exploited. Results of the present analyses point to a core set of fundamental residues, notably Y156 and Y157 (in *E. coli* and *S. aureus*, respectively) and A95, which have key interactions with different FabI inhibitors. The pair Y146/Y147 also have a potential for inhibitor binding, which is consistent with literature findings. The integration of MD simulations provides new perspectives regarding ligand–target complexes, revealing that certain residues located in more flexible regions, such as S197, might be less favorable for ligand binding due to their positional variability. Notably, our findings indicate a landscape of auxiliary binding sites, suggesting that residues such as I200, V201, and F203/F204 offer a promising and underexploited potential for designing novel inhibitors with enhanced efficacy and reduced susceptibility to resistance. Finally, by combining all experimental and computational structural data, we propose a reasonable pharmacophoric model for the FabI of both species, which might contribute to the VS of new potential FabI inhibitors and antibacterial drug candidates in the future.

## Author Contributions

P.T.T.F.L., L.H.S.O, F.K.M.O, M.S.M.S., T.K., and D.F.P. performed the in silico experiments and their respective data analyses, with support from V.G.M., G.C.V., and V.G.M. conceived the original idea, initiated the project, and oversaw all experiments and data analysis. P.T.T.F.L., L.H.S.O., T.K., P.O.F., and M.S.M.S. wrote the manuscript with assistance from other authors. All authors contributed to the manuscript writing, review, and provided comments and suggestions.

## Funding

T.K. is funded by the German Federal and State Governments and the German Center for Infection Research (DZIF, TTU06.716 and TTU 08.716). P.T.T.F.L. and G.C.V. are funded by the Coordenação de Aperfeiçoamento de Pessoal de Nível Superior (CAPES), and P.O.F. acknowledges the funding from Fundação de Amparo à Pesquisa do Estado de Minas Gerais (FAPEMIG). V.G.M. acknowledges the funding from the National Council for Scientific and Technological Development (CNPq, grant numbers 304958/2025‐5, 403811/2024‐4, and 443396/2024‐8) and FAPEMIG (grant number RED‐00110‐23). F.K.M.O. acknowledges her CAPES scholarship (88887.234483/2025‐00). A. M.‐G. thanks DGAPA, UNAM, Programa de Apoyo a Proyectos de Investigación e Innovación Tecnológica, for the Postdoctoral Fellowship. M.S.M.S. acknowledges his FAPEMIG fellowship (APD‐01212‐25).

## Conflicts of Interest

The authors declare no conflicts of interest.

## Supporting information

Supplementary Material

## Data Availability

The data that support the findings of this study are available from the corresponding author upon reasonable request.
