# Supplementary material for: Underexplored Ligand‐Binding Features of FabI From Staphylococcus aureus and Escherichia coli: A Comparative Pharmacophoric Modeling and Surface Mapping Approach
Source: ChemMedChem. 2026 Jul 31;21(15):e70410. doi: 10.1002/cmdc.70410 (PMC13425754; doi:10.1002/cmdc.70410)

Underexplored ligand-binding features of FabI from *Staphylococcus aureus* and *Escherichia coli*: a comparative pharmacophoric modeling and surface mapping approach

Pedro Tenório T. F. Leite,[a#] Lucas H. S. Ocarino,[a#] Gabriel C. Veríssimo,[a] Philipe O. Fernandes,[a] Dara Fernanda Pereira,[b] Fernanda Kelly M. e Oliveira,[c] Alberto Marbán-González,[d] José L. Medina Franco,[d] Mateus Sá M. Serafim,[c,a] Thales Kronenberger,[e,f,g*] Vinícius G. Maltarollo[a*]

[a] Departamento de Produtos Farmacêuticos, Faculdade de Farmácia, Universidade Federal de Minas Gerais (UFMG), Belo Horizonte, Minas Gerais 31270-901, Brazil.
[b] Centro Universitário UNA, Belo Horizonte, Minas Gerais, Brazil.
[c] Departamento de Análises Clínicas e Toxicológicas, Faculdade de Farmácia, Universidade Federal de Minas Gerais (UFMG), Belo Horizonte, Minas Gerais 31270-901, Brazil.
[d] DIFACQUIM Research Group, Department of Pharmacy, School of Chemistry, Universidad Nacional Autónoma de México, Avenida Universidad 3000, Mexico City 04510, Mexico.
[e] School of Pharmacy, Faculty of Health Sciences, University of Eastern Finland, 70211, Kuopio, Finland.
[f] Interfaculty Institute of Microbiology and Infection Medicine (IMIT), University of Tübingen, Tübingen, Germany.
[g] Partner-site Tübingen, German Center for Infection Research (DZIF), Tübingen, Germany.

[#] These authors equally contributed to this work.

[*]Corresponding authors: V.G.M, viniciusmaltarollo@gmail.com, Departamento de Produtos Farmacêuticos, Faculdade de Farmácia, Universidade Federal de Minas Gerais (UFMG), Belo Horizonte, Minas Gerais 31.270-901, Brazil. T.K., thales.kronenberger@uni-tuebingen.de Partner-site Tübingen, German Center for Infection Research (DZIF), DE-72076, Tübingen, Germany.

Support Information

Figure S1. Bemis & Murcko Scaffold diversity of the selected PDB entries used for pharmacophore modeling (**Table 1**).

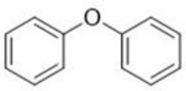

Scaffold 1
Freq. = 7

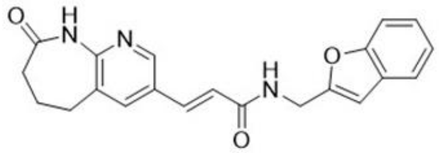

Scaffold 2
Freq. = 3

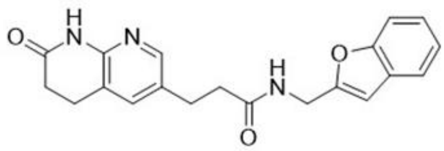

Scaffold 3
Freq. = 2

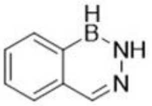

Scaffold 4
Freq. = 1

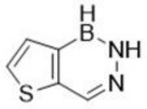

Scaffold 5
Freq. = 1

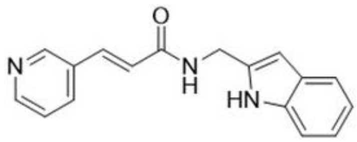

Scaffold 6
Freq. = 1

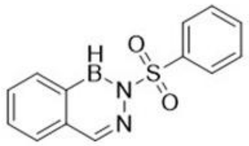

Scaffold 7
Freq. = 1

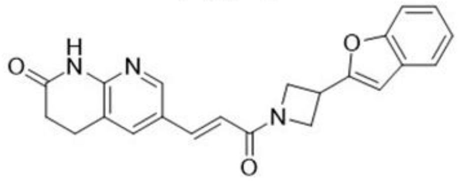

Scaffold 8
Freq. = 1

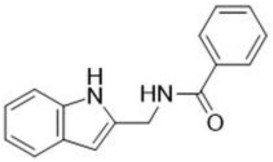

Scaffold 9
Freq. = 1

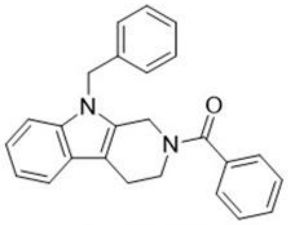

Scaffold 10
Freq. = 1

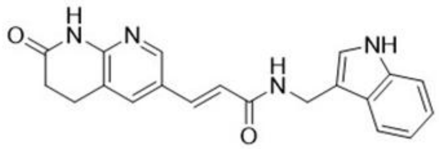

Scaffold 11
Freq. = 1

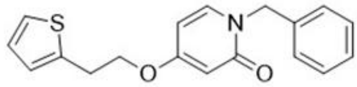

Scaffold 12
Freq. = 1

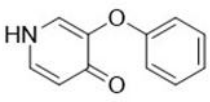

Scaffold 13
Freq. = 1

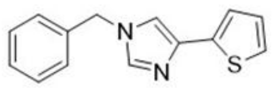

Scaffold 14
Freq. = 1

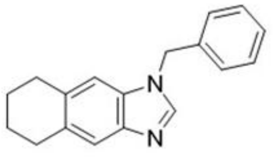

Scaffold 15
Freq. = 1

Figure S2. Multidimensional analyses based on structural alignment of all available crystals from FabI coloured by the selected crystals for pharmacophoric models vs MD selected frames (A), by species (B), by the crystal oligomerization states (C) and ligand binding class (D).

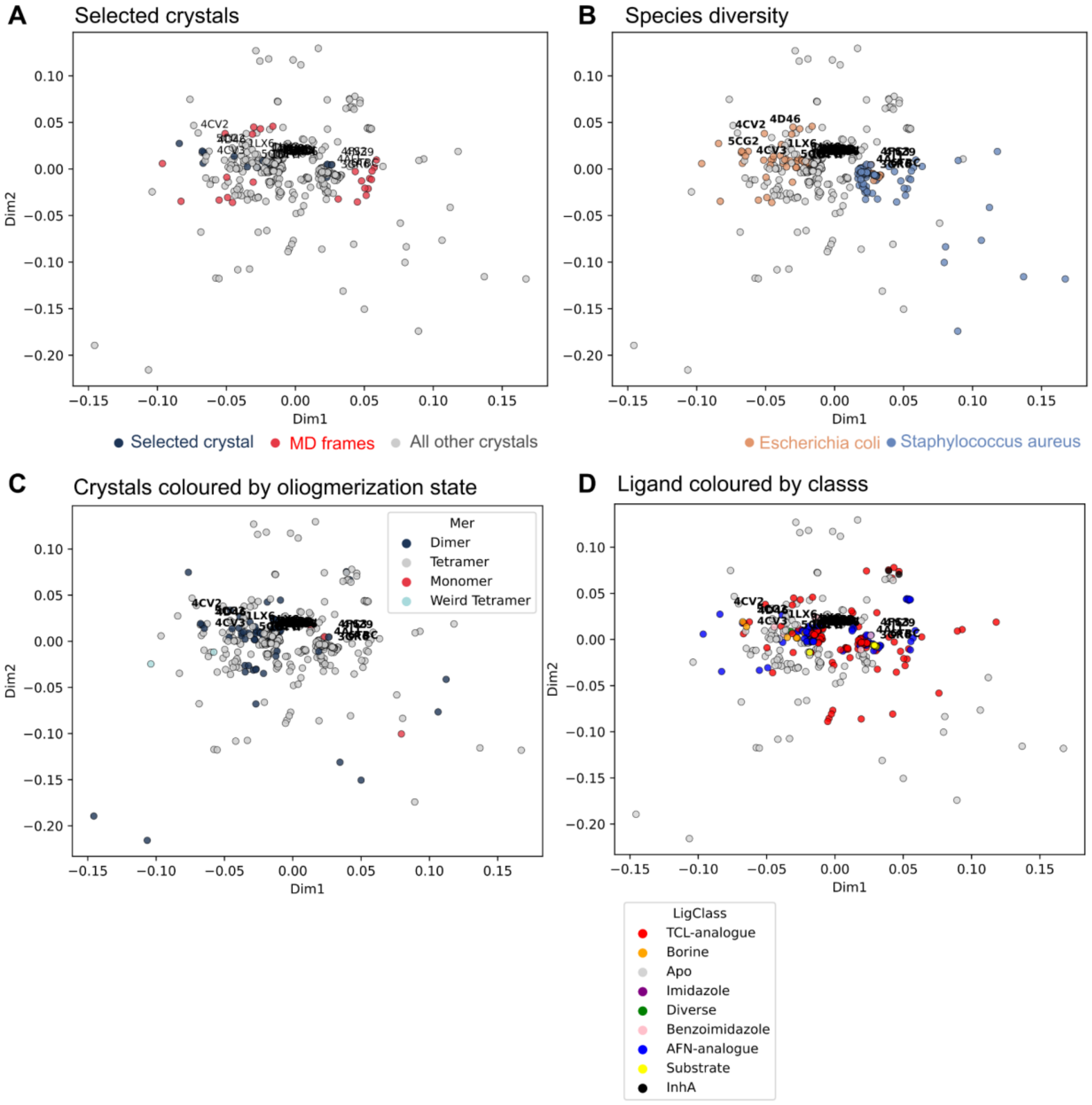

Figure S3. ROC curves of for *S. aureus* (A) and *E. coli* (B), for each vROCS output metric.

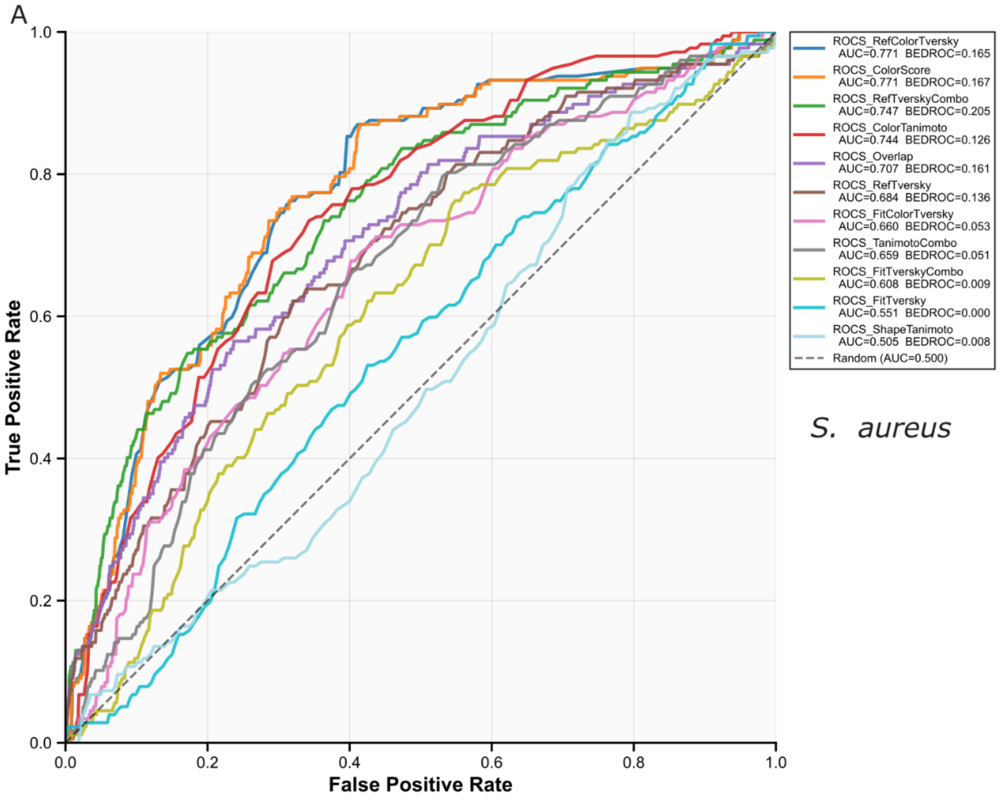

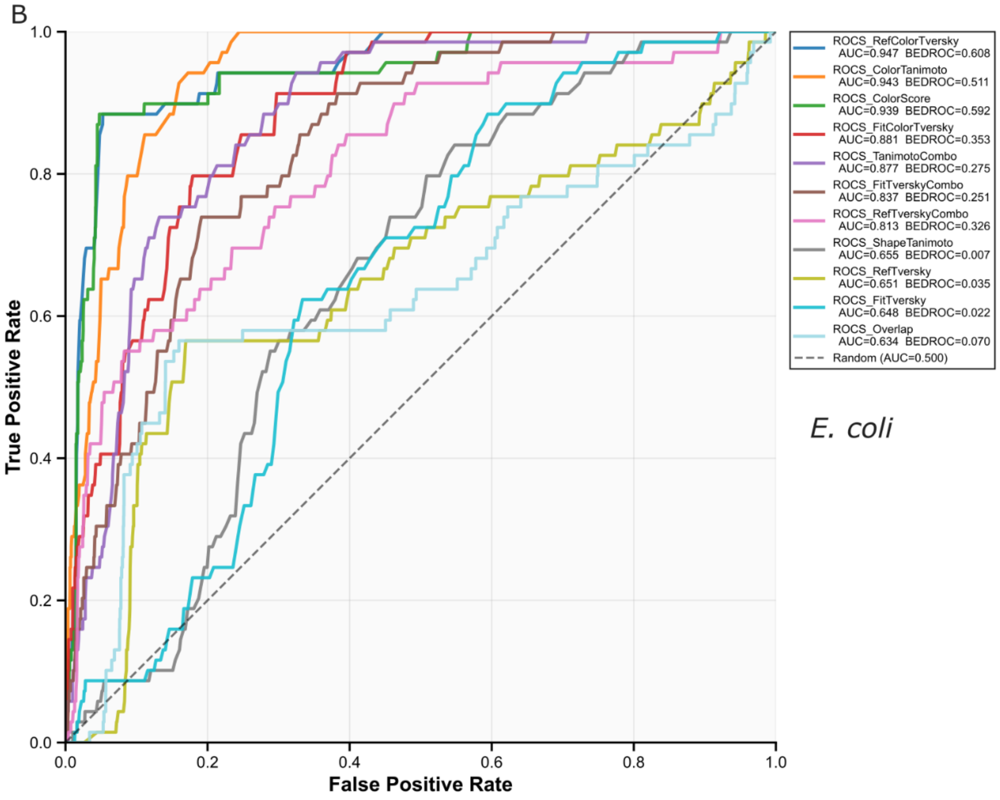

Figure S4. Chain A of crystal structure of *S. aureus* FabI in complex with ligand clusters obtained with FTMap. Clusters located on the cofactor pocket (yellow) were removed from further treatment, while clusters located on the inhibitor pocket (cyan) were kept.

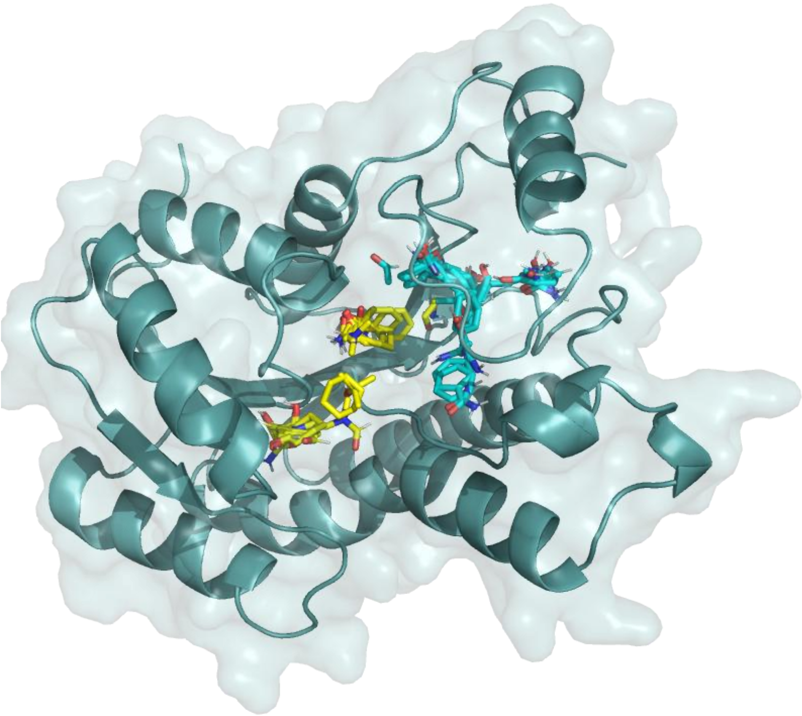

Figure S5. Residue interaction counts for each type of interaction in the S. *aureus* MD structures in complexes with TCL, MUT and ACT

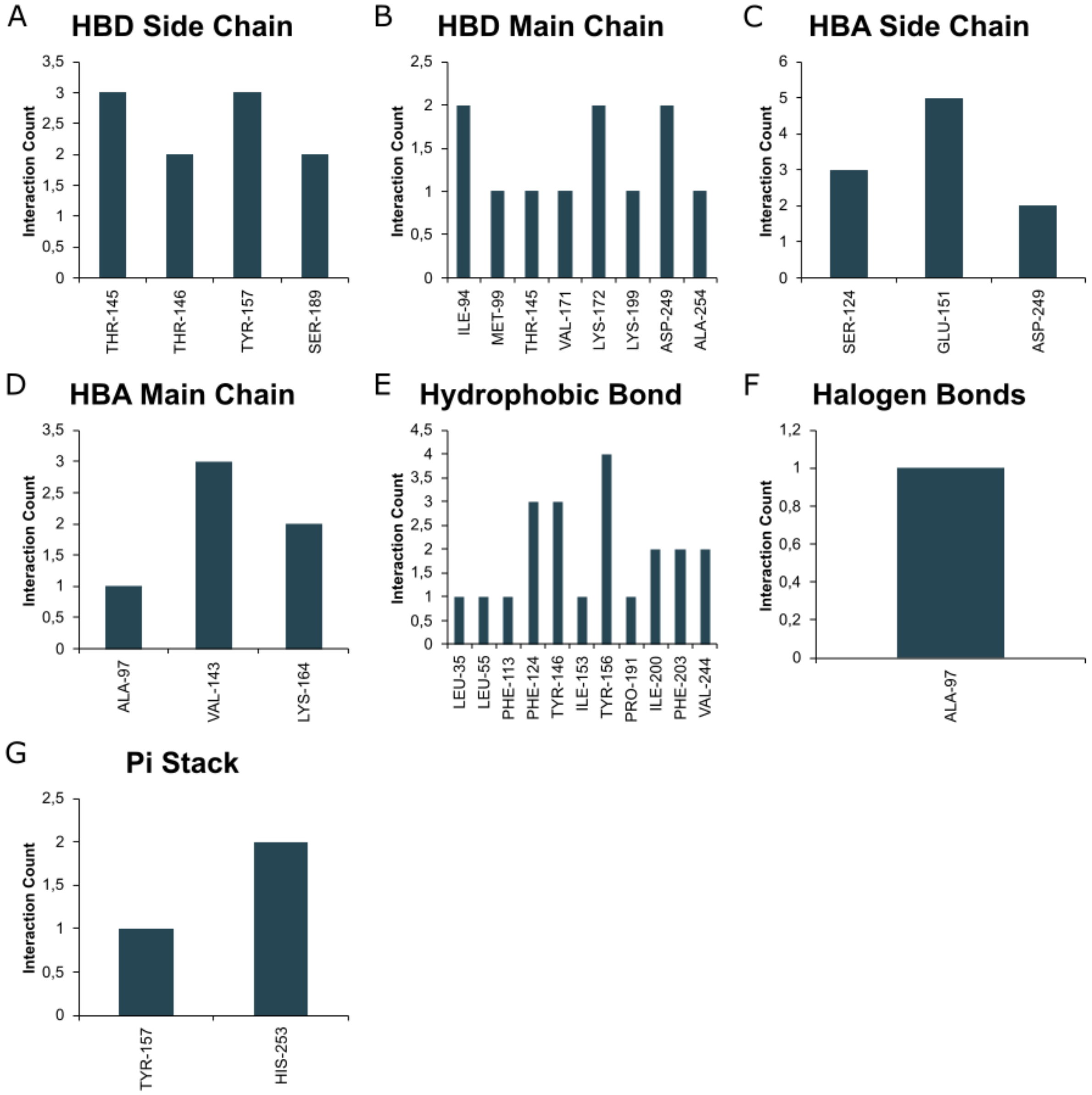

Figure S6. Residue interaction counts for the experimentally obtained S. *aureus* structures from PDB in complex with various ligands

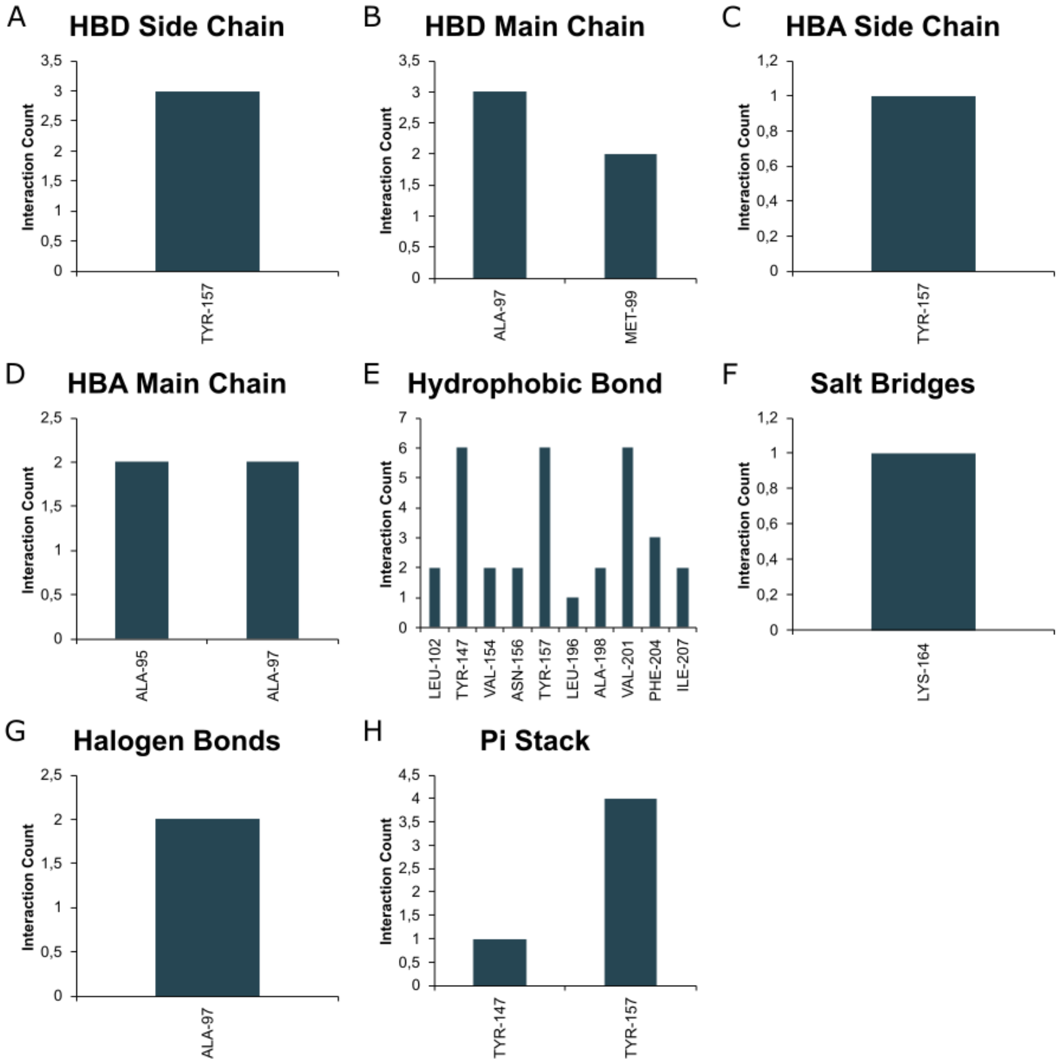

Figure S7. Residue interaction counts for each type of interaction in the E. *coli* MD structures in complexes with TCL, MUT and ACT

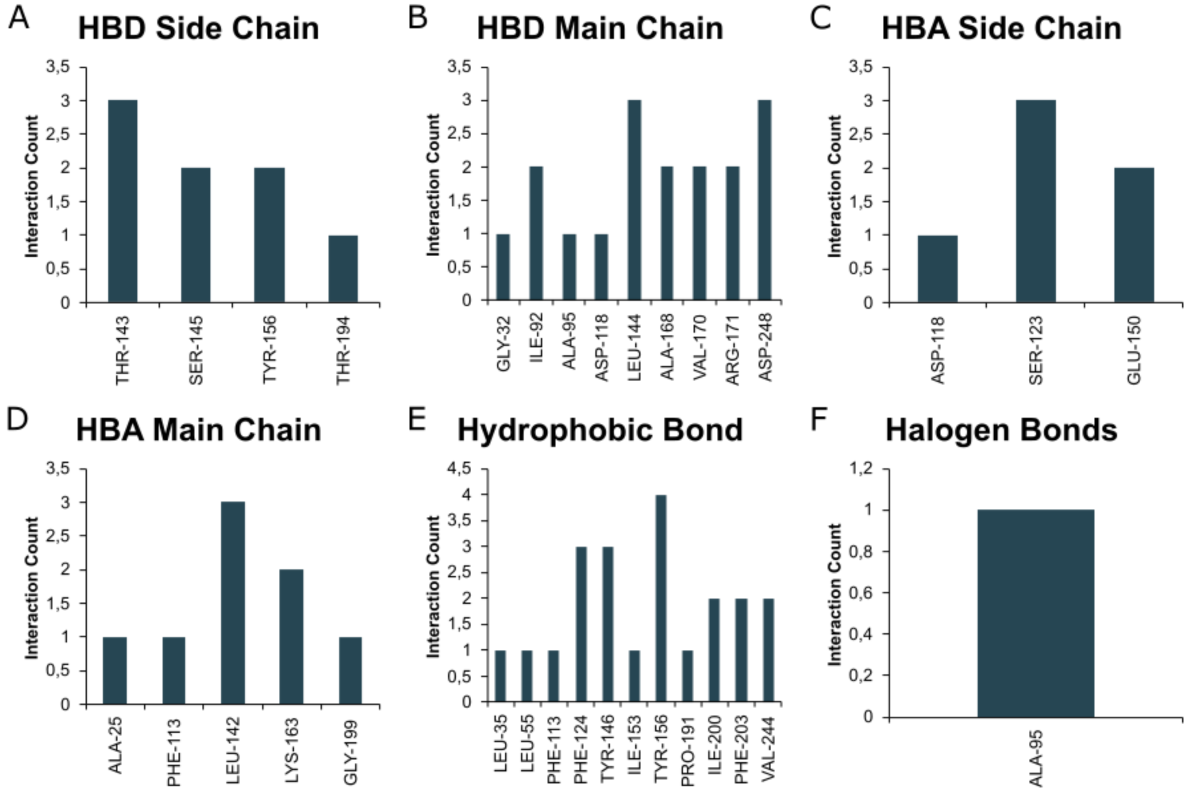

Figure S8. Residue interaction counts for the experimentally obtained E. *coli* structures from PDB in complex with various ligands

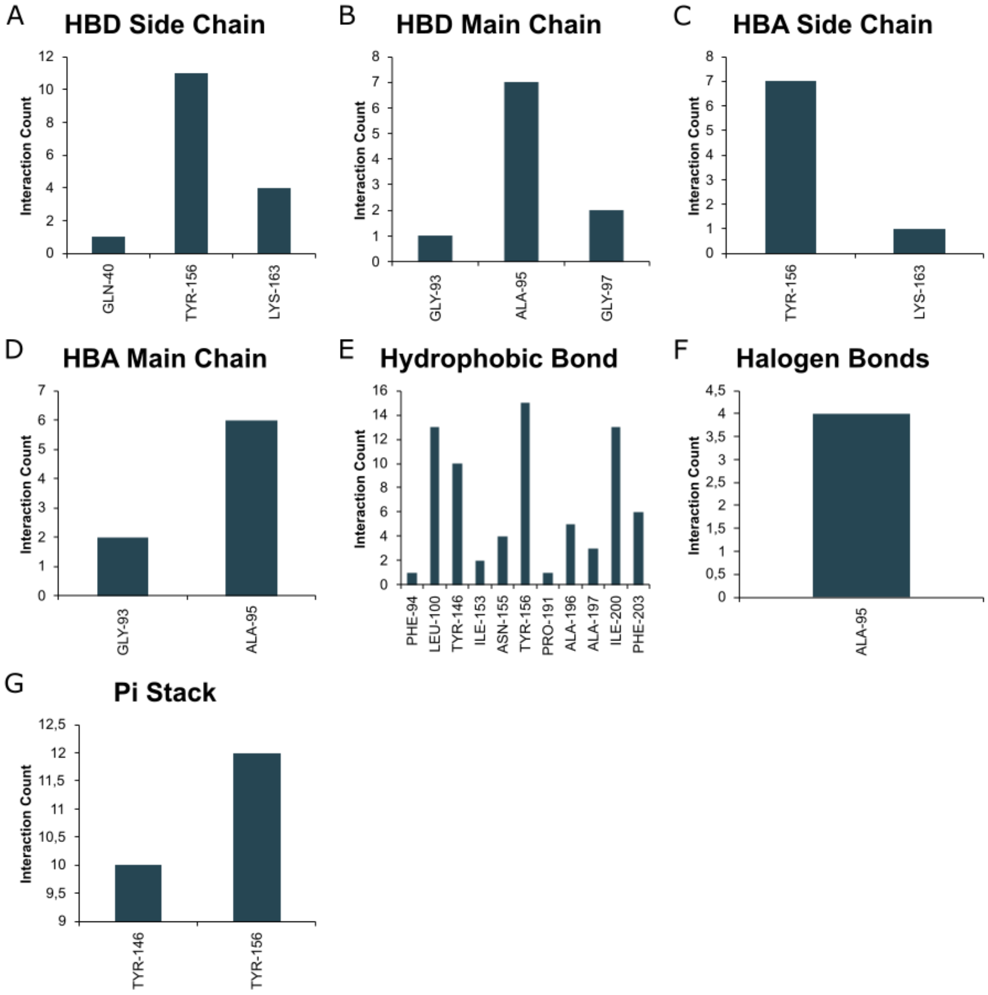

Figure S9. Electron density maps of TCL in 3GR6 (A) and 4ALL (B). The 2Fo–Fc map is shown as blue mesh contoured at 1.5σ and the negative Fo–Fc map as red mesh contoured at –3σ. Protein carbon atoms are dark blue, TCL carbon atoms green-cyan, and the cofactor light yellow.

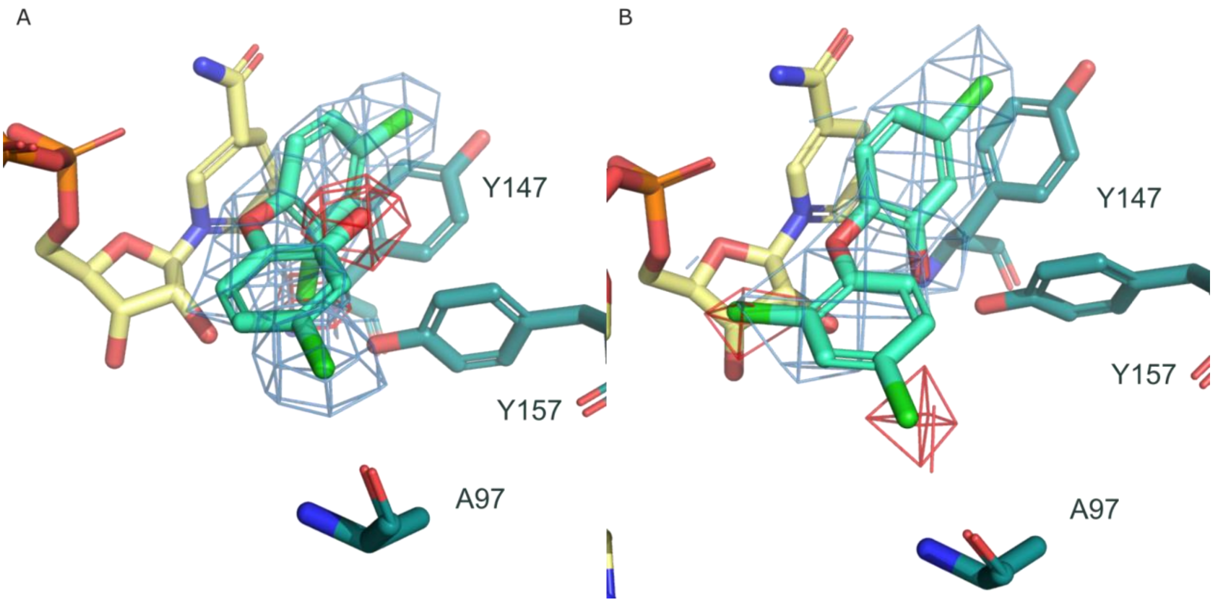

Supplement: Supplementary file 1 — Supplementary Material [file CMDC-21-e70410-s001.pdf]
